# The evaluation of the tests that can predict de novo stress urinary incontinence after advanced pelvic organ prolapse surgery: a prospective study

**DOI:** 10.1186/s12905-026-04687-6

**Published:** 2026-07-18

**Authors:** Reyhan Gündüz, Elif Ağaçayak

**Affiliations:** Department of Obstetrics and Gynecology, School of Medicine, Faculty of Medicine, Dicle University, Diyarbakır, Turkey

**Keywords:** Advanced POP, De novo stress urinary incontinence, Occult stress urinary incontinence, Reduced stress test, Urodynamics, Q-tip test

## Abstract

**Background:**

De novo stress urinary incontinence (SUI) may reduce patient satisfaction following a pelvic organ prolapse (POP) surgery and may necessitate additional anti-incontinence surgery in the future. The aim of this study is to evaluate physical examination tests that can predict de novo SUI after POP surgery.

**Methods:**

A total of 30 patients diagnosed with stage 3–4 POP who underwent POP surgery were included in this study. The results of the Q-tip test, reduced stress test, and urodynamic stress urinary incontinence test, which were performed before the surgery, and stress test results for SUI at 3 and 6 months after the surgery were recorded. The physical examination tests were compared and occult SUI and de novo SUI rates were determined.

**Results:**

Occult SUI detection rates were found to be 46.7% for the Q-tip test, 20% for the reduced stress test, and 3.3% for the urodynamic test. The rates of de novo SUI were determined as 3.3% and 10%, respectively, at the 3rd and 6th months postoperatively. The presence of gravida, parity, and the number of living children increased the detection rate of occult SUI by the Q-tip test at statistically significant level. It was also determined that the high number of abortions statistically increased the risk of de novo SUI at 6 months postoperatively and occult SUI did not significantly affect the risk of de novo SUI.

**Conclusion:**

Our findings suggest that less invasive and cost-effective physical examination tests may be considered in the preoperative evaluation of patients undergoing POP surgery; however, these results should be interpreted cautiously.

**Supplementary Information:**

The online version contains supplementary material available at https://doi.org/10.1186/s12905-026-04687-6.

## Introduction

Pelvic organ prolapse (POP) is defined as the pelvic organs (i.e., the uterus, bowel, bladder) descending down and as a result protruding out of the vagina [1]. POP is a common finding in the female population, occurring in approximately 50% of women who have given birth; however, only 10–20% of these women seek help for this problem [2]. POP can often be associated with various lower urinary tract symptoms such as stress urinary incontinence (SUI), urgency/urge urinary incontinence (UI), and voiding dysfunction/urinary retention [3].

SUI can be detected in 63% of women with POP. However, genital descensus may mask UI by flexing the urethra or by direct compression of the urethra [4]. In other words, some women with POP may not have UI because POP can increase pressure on the urethra, causing obstruction and maintaining continence. Only 33% of women with stage 4 POP have concomitant SUI. The UI can be unmasked by the reduction of the POP in 80% of cases. This type of UI is called “Occult SUI” and consists of demonstrable SUI in continent (i.e., asymptomatic) women after a reduction in POP. The term “de novo SUI” is used to describe SUI developing following surgical correction of the POP in women who were clinically continent before surgery. This may be because of the reduction of the urethral obstruction caused by POP, which may cause symptoms related to pre-existing urethral disruption [5, 6]. In this study, ‘occult SUI’ refers to stress urinary incontinence demonstrated only after prolapse reduction in previously continent women, and ‘de novo SUI’ refers to newly developed stress urinary incontinence following POP surgery.

Some authors recommend investigating occult SUI before surgery for POP because they believe it is associated with a higher risk for de novo SUI. The incidence of occult SUI is variable because there is no standard method of prolapse reduction, and some authors report rates of up to 80% [7]. The rates of de novo SUI after POP surgery are reported to be between 9.9% and 51% [5].

De novo SUI may reduce patient satisfaction following a POP surgery and may necessitate additional anti-incontinence surgery in the future. Prophylactic anti-incontinence surgery during POP surgery reduces the risk of de novo SUI [7, 8]; however, this approach has increased risks of surgical complications, and for this reason, it would be ideal to selectively apply prophylactic surgery only to women who have a high risk of de novo SUI. However, predictive testing for de novo SUI is not well known and clinicians still struggle to identify such patients [9, 10].

Preoperative evaluation for occult and de novo SUI includes anamnesis and physical examination. Physical examination tests may include an empty supine stress test, sitting or standing cough stress test (CST), pyridium pad test, and urodynamics. Studies were conducted to determine which test is best at detecting SUIs [11]. In general, these preoperative tests that have prolapse reduction have limitations because it was shown that a significant percentage of women who did not have incontinence at the time of preoperative testing still have postoperative SUI [12].

In this prospective study, the purpose was to evaluate the physical examination tests that can predict de novo SUI and to determine the risk factors by performing the preoperative physical examination tests on women with stage 3–4 POP who were continent before surgery. It was also aimed to contribute to the literature by determining the rates of occult SUI and de novo SUI in the women we included in the study.

## Material and method

A total of 30 patients who were diagnosed with Stage 3–4 POP who applied to the Dicle University Faculty of Medicine, Department of Obstetrics and Gynecology between August 2020 and October 2021 were included in this prospective study. The approval for the study was obtained from the Ethics Committee of Dicle University Faculty of Medicine on 16.07.2020 with the number 262. Written informed consent was obtained from each patient before inclusion in the study. The protocol was conducted in line with the Declaration of Helsinki Principles.

As no prospective study has previously evaluated and compared the predictive performance of the preoperative Q-tip test, reduced stress test, and urodynamic assessment for postoperative de novo stress urinary incontinence in women undergoing surgery for advanced pelvic organ prolapse, a moderate effect size (Cohen's d = 0.50) was adopted for the sample size estimation. The calculation was based on the primary endpoint, defined as the incidence of de novo stress urinary incontinence after surgery according to the preoperative test findings, using a two-tailed significance level of 0.05 and a statistical power of 80%. Based on these assumptions, at least 24 participants were required, and a total of 30 women were included in the final analysis.

Patients who did not have UI before surgery and had Stage 3–4 uterine prolapse according to POP-Q Classification and underwent prolapse surgery in our clinic were included in the present study. Patients who had previous incontinence surgery and POP surgery were not included in the study. Women who underwent physical examination the day before surgery and who underwent stress testing to evaluate SUI at 3 and 6 months postoperatively were also included in our study. Those with missing preoperative and postoperative physical examination test results were not included in the study and those with unregulated diabetes mellitus (DM), multiple sclerosis, Parkinson’s disease, and drug use (e.g., antihypertensive, beta-blocker, etc.) that might cause neurogenic incontinence were not included in the study. Only prolapse surgery was performed on all women, urinary incontinence surgery was not performed on any of the patients. Vaginal hysterectomy was combined with site-specific prolapse repair including anterior and/or posterior colporrhaphy and apical suspension according to the individual anatomical defect. All surgical procedures were performed in the same tertiary referral center according to standardized surgical principles routinely applied in our clinic.

The demographic data, body mass index (BMI) values, the results of the preoperative Q-tip test, reduced stress test, urodynamic stress incontinence test, and stress test for SUI in the 3rd and 6th months after the surgery were recorded for all patients who were included in the study. POP examination and physical examination tests of all patients were performed before and after the surgery by the same and only doctor working in our clinic.

A routine urogynecological evaluation was performed for each patient with anamnesis and routine urogynecological examinations. Urinalysis and urine culture were performed before the physical examination tests were conducted, and the patients who were found to be infected were treated. Physical examination tests (Q-tip test, reduced stress test, and urodynamics), the prolapse was performed by reduction with a gauze pad held by forceps in a semisupine lithotomy position and the bladder was approximately 200 ml full. The diagnosis of occult SUI was initially assessed with a reduced stress test. Urine leakage from the external urethral meatus during coughing and straining was accepted as a positive stress test (i.e., occult SUI). The mobility of the bladder neck was evaluated with the Q-tip test. Angling of the cotton swab inserted from the urethral meatus was observed at rest and in the presence of approximately 200 mL of urine in the bladder. If the angle was more than 35°, it was accepted that the anatomical support of the bladder neck decreased and the mobility increased, and the test result was evaluated as occult SUI [13]. Urodynamics was performed with a multi-channel air system (Solar Uro model, MMS/LABORIE, The Netherlands). Urodynamic stress incontinence at maximal bladder capacity was diagnosed when urine leakage was detected without a simultaneous increase in detrusor pressure during coughing or Valsalva Maneuver [14]. The cough stress test was performed in the semisupine lithotomy position while the bladder was comfortably filled with at least 200 mL of urine in the 3rd and 6th months after the surgery. Postoperative de novo SUI was defined as newly developed urine leakage observed during the postoperative cough stress test in previously continent women. However, pad test quantification, leakage volume measurement, and validated patient-reported outcome measures were not included in the study due to study design and resource limitations.

The conformity of the numerical variables to the normal distribution was tested with the Shapiro–Wilk test. The student’s ***t***-test was used to compare the normally distributed variables in two groups, and Mann–Whitney U test was used to compare the non-normally distributed variables in two groups. Relationships between categorical variables were evaluated using the Chi-square test or Fisher’s exact test, as appropriate. The McNemar’s test was used to evaluate two-category dependent measures and the SPSS 22.0 Windows version package program and MedCalc 19.7.1 package program were used in the analysis. *P* < 0.05 was considered statistically significant.

## Results

The mean age of the women who were included in this study was found to be 61.5 (40- 80) years, the number of gravida was 8 (1–20), the mean BMI score was 28.5 (20.5–44.1) kg/m^2^, 22 (73.3%) patients had Stage 3 POP, and 29 (96.7%) patients underwent vaginal hysterectomy (Table 1).

**Table 1:** Evaluation of demographic data and clinical values

	**Median (Min—Max)** **(*n* = 30)**	
Age	61.5 (40–80)	
Gravida	8 (1–20)	
Parity	7 (1–16)	
Abortion	0 (0–5)	
Living	6 (1–14)	
Height (cm)	156 (141–165)	
Weight (kg)	70.5 (43–105)	
BMI (kg/m^2^)	28.55 (20.5–44.1)	
		**n (%)**
POP Stage	Stage III	22 (73.3)
	Stage IV	8 (26.7)
Type of surgery	Le Forte	1 (3.3)
	Vaginal hysterectomy	29 (96.7)

The detection rates of occult SUI in the physical examination tests were found to be 46.7% (14) in the Q-tip test, 20% (6) in the reduced stress test, and 3.3% (1) in the urodynamic test. The rates of de novo SUI in the postoperative 3rd and 6th months were 3.3% (1) and 10% (3), respectively (Table 2).

**Table 2:** Occult SUI detection rates and de novo SUI rates in the physical examination tests

		**n (%)**
Q-tip test occult SUI	Positive	14 (46.7)
	Negative	16 (53.3)
Reduced stress test occult SUI	Positive	6 (20)
	Negative	24 (80)
Occult SUI in urodynamics	Yes	1 (3.3)
	No	29 (96.7)
De novo SUI at 3-month follow-up	Yes	1 (3.3)
	No	29 (96.7)
De novo SUI at 6-month follow-up	Yes	3 (10)
	No	27 (90)

Considering the factors increasing the risk of occult SUI in physical examination tests, a statistically significant difference was found regarding the presence of gravida, parity, and the number of living children, which increased the detection rate of occult SUI by the Q-tip test (*p* < 0.05). No statistically significant factors were detected in the detection rate of occult SUI with the reduced stress test (*p* > 0.05) (Table 3). It was also found that the high number of abortions increased the risk of de novo SUI statistically in the postoperative 6th month (*p* < 0.05) and other variables had no effects on the risk of de novo SUI in the postoperative 6 months (*p* > 0.05) (Table 4). The POP stage did not affect the risk of occult SUI in both the Q-tip test or the reduced stress test and the risk of de novo SUI in the 6th postoperative month (*p* > 0.05).

**Table 3:** Evaluation of factors affecting occult SUI in patients with occult SUI detected with the Q-tip test and reduced stress test

	**Occult SUI with Q-tip test**			**Occult SUI with reduced stress test**		
	**Positive (*n* = 14)** **Median (Min—Max)**	**Negative (*n* = 16)** **Median (Min—Max)**	**p**	**Positive** **(*n* = 6)** **Median (Min—Max)**	**Negative (*n* = 24)** **Median (Min—Max)**	**p**
Age	63.43 ± 8.31	58.81 ± 10.04	0.185ǂ	60.33 ± 7.15	61.13 ± 10.02	0.940Ɨ
Gravida	10.07 ± 4.01	6.38 ± 3.26	0.010*ǂ	6 ± 2.9	8.63 ± 4.15	0.157ǂ
Parity	8.71 ± 3.07	5.31 ± 2.7	0.010*Ɨ	4.67 ± 2.5	7.46 ± 3.3	0.064ǂ
Abortion	1.36 ± 1.45	0.94 ± 1.69	0.334Ɨ	1.33 ± 2.16	1.08 ± 1.44	0.860Ɨ
Living	7.79 ± 2.94	4.63 ± 2.36	0.003*ǂ	4.17 ± 1.94	6.58 ± 3.12	0.083ǂ
BMI	30.8 ± 5.25	28.47 ± 4.98	0.222ǂ	28.93 ± 8.75	29.71 ± 4.09	0.839ǂ

^*^ Significant at the *p* < 0.05 level, ǂ Student *t*-Test. Ɨ Mann–Whitney U test

**Table 4:** Evaluation of the factors affecting de novo SUI in postoperative 6 months

	**Yes (*n* = 3)**	**No (*n* = 27)**	**p**
Age	66.33 ± 3.06	60.37 ± 9.72	0.226
Gravida	9.67 ± 2.52	7.93 ± 4.16	0.387
Parity	6.67 ± 4.16	6.93 ± 3.3	0.795
Abortion	3 ± 1.73	0.93 ± 1.44	0.041*
Living	6.67 ± 4.16	6.04 ± 3.01	0.647
BMI	25.9 ± 3.44	29.96 ± 5.2	0.226

^*^Significant at the *p* < 0.05 level, Mann–Whitney U test

It was found in the present study that occult SUI detected in the physical examination test results (Q-tip test, reduced stress test, and urodynamics) did not have any statistical effects on the risk of de novo SUI occurring in the postoperative 3rd and 6th months (*p* > 0.05) (Table 5).

**Table 5:** The effect of patients with occult SUI detected with the Q-tip test, reduced stress test and urodynamics on the risk of de novo SUI

		**De novo SUI at 3-month follow-up**		**De novo SUI at 6-month follow-up**	
		**Yes**	**No**	**Yes**	**No**
Q-tip test occult SUI	Positive	1 (7.1)	13 (92.9)	2 (14.3)	12 (85.7)
	Negative	0 (0)	16 (100)	1 (6.3)	15 (93.8)
	p	0.467		0.586	
Reduced stress test occult SUI	Positive	0 (0)	6 (100)	1 (16.7)	5 (83.3)
	Negative	1 (4.2)	23 (95.8)	2 (8.3)	22 (91.7)
	p	1.000		0.501	
Occult SUI in urodynamics	Positive	0 (0)	1 (100)	0 (0)	1 (100)
	Negative	1 (3.4)	28 (96.6)	3 (10.3)	26 (89.7)
	p	1.000		1.000	

Significant at *p* < 0.05 level, Fisher’s exact test

It was found that the postoperative follow-up period (3rd and 6th months) did not make a statistically significant difference in de novo SUI rates (*p* > 0.05) (Table 6).

**Table 6:** Comparison of de novo SUI rates at 3- and 6-month follow-up

		**De novo SUI at 3-month follow-up**		
		**Yes**	**No**	
		**n (%)**	**n (%)**	**p**
De novo SUI at 6-month follow-up	Yes	1 (100)	2 (6.9)	0.500
	No	0 (0)	27 (93.1)	

Significant at *p* < 0.05 level, McNemar’s test

## Discussion

Surgical management of POP patients without preoperative UI scheduled for surgery is not certain. Postoperative de novo SUI is a concern for the surgeon and the patient and creates the need for additional anti-incontinence surgery in the future. The addition of anti-incontinence surgery to the POP surgery of women who were continent before surgery is controversial. For this reason, it is important to find the best physical examination test that can predict de novo SUI. In this study, the purpose was to perform the reduced stress test, Q-tip test, and urodynamic test on patients before POP Surgery and to determine the rate of these physical examination tests to detect occult and de novo SUI and the risk factors for de novo SUI. Although occult SUI was frequently detected, no statistically significant association with de novo SUI was observed in our cohort. However, this finding should be interpreted cautiously due to the small sample size, low number of outcome events, and short follow-up period. As a result of the study, it was found that the occult SUI rates were 46.7%, 20%, and 3.3% for Q-tip test, reduced stress test, and urodynamic tests, respectively, and de novo SUI rates were 3.3% and 10%, respectively at the postoperative 3rd and 6th months. Our findings did not demonstrate a statistically significant association between occult SUI and de novo SUI, and the postoperative follow-up period (3rd and 6th months) is not significantly different for de novo SUI. One possible explanation for this finding is the limited statistical power of the study. A true association may not have been detected. In addition, multivariate analysis could not be performed due to the small sample size and low number of outcome events, which limited the ability to control for potential confounding factors.

In their study conducted with 274 patients without occult SUI in the preoperative continent and urodynamics who are scheduled POP surgery, Alas et al. reported the rate of de novo SUI as 9.9% in patients without occult SUI. Since the incidence of de novo SUI was found to be low, it was not recommended to add concomitant anti-incontinence surgery to POP surgery [5]. In their study, Martin et al. included 84 patients with Stage 2 and above POP and planned for POP surgery and reported that 64.3% of the patients were Stage 3 and 2.4% Stage 4 POP, and they performed preoperative urodynamics, stress test, and 24-h pad test. The rate of occult SUI was reported to be 42.5%, 17.5%, and 10% for urodynamics, stress test, and 24-h pad test, respectively. They also concluded that the sensitivities of stress and pad testing for stress incontinence and occult SUI in patients with POP were low compared to urodynamics as criteria [15]. In the study of Hwang et al., 105 patients with Stage 3–4 POP were included. Occult SUI rate was reported as 60%, but 25.7% of them were stated to be continent. The sensitivities for detecting occult SUI with physical examination (with and without reduction of stress test) and urodynamics were 88.9% and 87.3%, respectively. It was concluded that physical examination tests and urodynamics were equally effective, and it was necessary to use urodynamics to obtain more information on the lower urinary system [16]. Pecchio et al. conducted a retrospective study including 155 patients with Stage 2 and above POP who underwent POP surgery and reported that 94 patients were preoperatively continent. It was also stated that the rate of occult SUI was 36% and the rate of de novo SUI was 16%, and it was recommended to perform two-stage surgery to reduce surgical complications in patients whose surgical method was preoperatively continent [17]. When we compared our results with the study conducted by Alas et al., the de novo rate was found to be 3.4% and 10.3%, respectively, in the 3rd and 6th months postoperatively in patients without occult SUI in urodynamics. In other words, although occult SUI is not detected in preoperative physical examination tests, we must keep in mind that de novo SUI might be present. In the present study, occult SUI rates were found to be 46.7%, 20%, and 3.3% for Q-tip test, reduced stress test, and urodynamic tests, respectively, and de novo SUI rates were 3.3% and 10%, respectively, at the 3rd and 6th months postoperatively. There is no accurate and standardized diagnostic test to detect occult SUI and de novo SUI. When the studies in the literature were examined to evaluate the detection rates of tests for occult SUI and de novo SUI predictivity, different rates were detected. To minimize these differences and increase the reliability of the present study, we paid attention to the fact that all the patients included were preoperative continent patients, we performed the prolapse reduction with the same method when performing the preoperative physical examination tests, and by performing only POP surgery on the patients, an objective comparison was possible in the preoperative tests for all postoperative patients.

Cohn et al. concluded that de novo SUI might develop following the prolapse repair in approximately half of the women who did not have clinical SUI, an increased risk among women with occult SUI, or anamnesis of preoperative SUI. As a surgical option, it was recommended to evaluate the risk for de novo SUI and choose according to the patient's preference [18]. In their study conducted with 299 patients who were preoperatively continent were included, Ugianskiene et al. reported that de novo SUI developed in 6% of the patients in the postoperative 3rd month, de novo SUI decreased in long-term follow-ups, and a high number of parity was a risk factor for de novo UI. If POP surgery is to be performed on continent women, it was recommended not to add concomitant anti-incontinence surgery to POP surgery because the risk of de novo UI is low [19]. In the study conducted by Lo et al., only women who were clinically continent before surgery and showed no signs of stress urinary incontinence on preoperative urodynamic testing were included. The de novo SUI rate was reported as 11% in the postoperative 6-month and 1-year follow-up. As a result, it was reported that the risk of de novo SUI was higher in women over 66 years of age with advanced POP and DM, and such patients must be evaluated for adding anti-incontinence surgery together with POP surgery [20]. When another study in the literature that discussed the risk factors for de novo SUI was evaluated, patients who were continent preoperatively and had only POP surgery were followed up for one year postoperatively. The de novo SUI rate was reported to be 9%. It was also stated that the predictive factors for de novo SUI were limited, and the risk of de novo SUI development was 7 times higher in the 12th month postoperatively in patients with preoperative occult SUI [21]. However, Manodoro et al. conducted a study with 150 patients who had only POP surgery followed up for an average of 18 months postoperatively. In urodynamic terms, it was stated that occult SUI had a weak predictivity for de novo SUI [4]. In this study, the risk factors for both occult SUI and de novo SUI were evaluated separately. We found no risk factor for occult SUI in the reduced stress test, and gravida, parity, and the high number of living children were risk factors for occult SUI in the Q-tip test. It was also determined that a high number of abortions is a risk factor for patients with de novo SUI at 6 months postoperatively. There was no significant relationship between occult SUI detected in preoperative tests and de novo SUI, and there was no significant difference for de novo SUI between the 3rd and 6th month postoperative follow-up period. As mentioned before, the choice of surgical treatment for POP patients is controversial. The issue of which anti-incontinence surgery must be added to POP surgery is still seen as an incomplete topic in the literature because there is no standard test to determine de novo SUI and no standard for the reduction method applied to perform the test. Also, the difference in POP surgery may change the risk of de novo SUI. Even POP surgery can sometimes act like anti-incontinence surgery. The development of de novo SUI after POP surgery is a clinical complication. However, to avoid this complication, performing anti-incontinence surgery on every patient who is scheduled for POP surgery may lead to additional surgical complications for patients who will not develop de novo SUI. For this reason, when the results of our study and other studies in the literature were evaluated, we think that adding anti-incontinence surgery to POP surgery in preoperative continent patients may be overtreatment because we do not yet know a reliable test to predict de novo SUI. Comprehensive studies with a larger number of patients are needed in this respect.

The strengths of the present study include that urodynamic and physical examination tests were performed by the same person and with the same method. The study was prospective and consisted of patients of the same ethnic origin. Another advantage is that all patients included in the study had advanced-stage prolapse with a strict methodology and had no SUI complaints, and only POP surgery was performed on all participants. Unlike previous reports, this study assessed occult SUI and postoperative de novo SUI as distinct outcomes, with evaluations performed at both 3 and 6 months following surgery. The limitations of the study include the small sample size, the short postoperative follow-up period, and the lack of additional outcome measures such as pad test quantification, leakage volume assessment, and patient-reported symptom evaluation, which may limit the comprehensive assessment of de novo SUI. Importantly, only a small number of patients developed de novo SUI during follow-up, which limited the statistical power and may have reduced the ability to detect significant associations. Given the limited number of outcome events, the results of this study should be interpreted as exploratory rather than definitive. Furthermore, surgical variables were not analyzed in detail in this study and may have influenced postoperative outcomes, representing a potential source of bias.

## Conclusion

In this prospective exploratory study, the presence of occult SUI identified by preoperative assessments was not significantly associated with the development of postoperative de novo SUI. Nevertheless, the relatively small sample size and the low number of outcome events limit the strength of these findings. Therefore, larger prospective studies are needed to validate these results before definitive conclusions can be established.

## Supplementary Information

Supplementary Material 1.

## Data Availability

The datasets used and/or analysed during the current study are available from the corresponding author on reasonable request.

## Abbreviations

BMI: Body Mass Index
CST: Cough Stress Test
DM: Diabetes Mellitus
POP: Pelvic Organ Prolapse
POP-Q: Pelvic Organ Prolapse Quantification
SUI: Stress Urinary Incontinence
UI: Urinary Incontinence
