# Supplementary material for: The evaluation of the tests that can predict de novo stress urinary incontinence after advanced pelvic organ prolapse surgery: a prospective study
Source: BMC Womens Health. 2026 Jul 18;26:524. doi: 10.1186/s12905-026-04687-6 (PMC13663212; doi:10.1186/s12905-026-04687-6)
Supplement: Supplementary file 1 [file 12905_2026_4687_MOESM1_ESM.docx]

| **STROBE Item** | **Recommendation** | **Reported on Page** |
| --- | --- | --- |
| Title and abstract | Study design indicated in title/abstract | Pages 1–2 |
| Background/rationale | Scientific background explained | Pages 3–4 |
| Objectives | Study objectives stated | Page 4 |
| Study design | Prospective study described | Page 5 |
| Setting | Dates and setting described | Page 5 |
| Participants | Inclusion/exclusion criteria reported | Page 5 |
| Variables | Occult SUI and de novo SUI defined | Pages 3, 6 |
| Data sources/measurement | Q-type, reduced stress, urodynamics explained | Page 6 |
| Bias | Limitations and possible bias discussed | Page 12 |
| Study size | Power analysis reported | Page 5 |
| Quantitative variables | Statistical handling described | Page 7 |
| Statistical methods | Statistical analyses detailed | Page 7 |
| Participants/results | Participant numbers and outcomes reported | Pages 7–8 |
| Descriptive data | Demographics and clinical data shown | Table 1 |
| Outcome data | Occult and de novo SUI rates reported | Table 2 |
| Main results | Statistical findings reported | Tables 3–6 |
| Key results | Summary of findings | Pages 8–12 |
| Limitations | Study limitations discussed | Page 12 |
| Interpretation | Interpretation of findings provided | Pages 8–12 |
| Generalisability | Need for larger studies acknowledged | Page 12 |
| Funding | Funding source reported | Page 1 |
|  |  |  |
